# Mitochondrial Fragmentation in a High Homocysteine Environment in Diabetic Retinopathy

**DOI:** 10.3390/antiox11020365

**Published:** 2022-02-11

**Authors:** Renu A. Kowluru, Ghulam Mohammad

**Affiliations:** 1 Department of Ophthalmology, Visual and Anatomical Sciences, Wayne State University, Detroit, MI 48201, USA; ed5563@wayne.edu; 2 Kresge Eye Institute, Wayne State University, Detroit, MI 48201, USA

**Keywords:** diabetic retinopathy, dynamin-related protein 1, homocysteine, mitochondrial dynamics, nitrosylation, homocysteine, mitochondrial fission and diabetic retinopathy

## Abstract

Diabetic patients routinely have elevated homocysteine levels, and due to increase in oxidative stress, hyperhomocysteinemia is associated with increased mitochondrial damage. Mitochondrial homeostasis is directly related to the balance between their fission and fusion, and in diabetes this balance is disturbed. The aim of this study was to investigate the role of homocysteine in mitochondrial fission in diabetic retinopathy. Human retinal endothelial cells, either untransfected or transfected with siRNA of a fission protein (dynamin-related protein 1, Drp1) and incubated in the presence of 100 μM homocysteine, were analyzed for mitochondrial fragmentation by live-cell microscopy and GTPase activity of Drp1. Protective nucleoids and mtDNA damage were evaluated by SYBR DNA stain and by transcripts of mtDNA-encoded *ND6* and *cytochrome b*. The role of nitrosylation of Drp1 in homocysteine-mediated exacerbation of mitochondrial fragmentation was determined by supplementing incubation medium with nitric-oxide inhibitor. Homocysteine exacerbated glucose-induced Drp1 activation and its nitrosylation, mitochondrial fragmentation and cell apoptosis, and further decreased nucleoids and mtDNA transcription. *Drp1*-siRNA or nitric-oxide inhibitor prevented glucose- and homocysteine-induced mitochondrial fission, damage and cell apoptosis. Thus, elevated homocysteine in a hyperglycemic environment increases Drp1 activity via increasing its nitrosylation, and this further fragments the mitochondria and increases apoptosis, ultimately leading to the development of diabetic retinopathy.

## 1. Introduction

Retinopathy is one of the most feared complications of diabetes, and, despite extensive pioneering research in the field, it still remains the leading cause of blindness in working-aged adults [1]. Oxidative stress is increased in diabetes, the mitochondrial structure and DNA are damaged in the retina and its vasculature, and mitochondrial function is impaired. Mitochondrial dysfunction is intimately associated with the development of diabetic retinopathy [2,3,4]. Mitochondria are highly dynamic organelles undergoing coordinated cycles of fission and fusion, referred as ‘mitochondrial dynamics’. This helps them maintain their shape, size and distribution, which are crucial for their quality control and other cellular processes, such as apoptosis. While fusion results in the formation of a single mitochondrion by mixing the contents of previously independent, partially damaged mitochondria, fission forms new mitochondria [5]. In diabetic retinopathy, while fusion protein mitofusin 2 (Mfn2) is downregulated, fission GTPase dynamin-related protein 1 (Drp1) is upregulated, leading to increased mitochondrial fission [6,7]. Although fission is important in segregating the damaged mitochondria for degradation, excessive fission is linked to apoptosis [8], retinal capillary cells undergo accelerated apoptosis, which precedes the development of histopathologic characteristic of diabetic retinopathy [9,10]. Drp1 is mainly a cytosolic protein, and it translocates to the mitochondrial outer membrane to initiate the fission process. Mitochondrial translocation of Drp1 and its GTPase activity are closely dependent on its interaction with an outer membrane fission factor 1 [11]. Once on the mitochondrial membrane, Drp1 helps to form a ring-like structure around the mitochondria, which narrows the membrane to help them divide. Disruption in the balance between mitochondrial fission and fusion leads to mitochondrial dysfunction [12,13].

Although hyperglycemia is considered the main integrator, many systemic factors, such as hyperlipidemia and hypertension, are also now implicated in the development of diabetic retinopathy [14]. Diabetic patients have elevated levels of a nonprotein amino acid, homocysteine [15], and high homocysteine is associated with increased oxidative stress and endothelial cell dysfunction in many vascular diseases, including cardiovascular disease and diabetic retinopathy [16,17]. Retinas from human donors with established diabetic retinopathy also have increased homocysteine levels compared to their age-matched, nondiabetic, human donors, and hyperglycemia in a hyper-homocysteinemic milieu produces additive damage to the retinal vasculature [7,18]. Homocysteine is shown to damage the mitochondrial fusion–fission process in the mesenteric artery, leading to endothelial cell damage [19], and retinal ganglion cells from mice deficient in cystathionine-β-synthase, an enzyme critical for the conversion of homocysteine to cysteine, have increased mitochondrial fission [20]. The role of homocysteine in mitochondrial dynamics in diabetic retinopathy is unclear.

Post-translational modifications are considered to play an important role in the subcellular localization of Drp1; by altering Drp1 binding with the receptor proteins, these modifications affect its functional properties [21]. Protein S-nitrosylation, a prominent redox reaction mediating nitric oxide (NO) signaling, is a ubiquitous post-translational modification that is responsible for a broad spectrum of biological functions [22]. Nitrosylation of Drp1 is shown to facilitate its activation [23], and in diabetes, retinal NO production is elevated [24]. Moreover, a NO-dependent mechanism is implicated in homocysteine-induced apoptosis in trigeminal sensory neurons [25]. Whether Drp1 nitrosylation has any role in mitochondrial fragmentation in diabetic retinopathy, and the presence of homocysteine further affects Drp1 nitrosylation, remain elusive.

The aim of this study was to investigate the role of homocysteine in Drp1-mediated mitochondrial fragmentation in diabetic retinopathy. Using human retinal endothelial cells (HRECs) in culture, effect of homocysteine on Drp1-mediated mitochondrial fragmentation was determined. To understand the mechanism of Drp1 activation, effect of homocysteine on its nitrosylation was evaluated.

## 2. Methods

Endothelial cells from human retinas (HRECs, Cat. No. ACBRI 181, Cell Systems Corp., Kirkland, WA, USA) were cultured in Dulbecco’s Modified Eagle Medium (Cat no. D5523, Sigma-Aldrich, St. Louis, MO, USA) containing 12% heat-inactivated fetal bovine serum, 15 μg/mL endothelial cell growth supplement and 1% each of insulin, transferrin, selenium, glutamax and antibiotic/antimitotic. Cells were grown at 37 °C in a humidified environment of 95% O_2_ and 5% CO_2_, and confluent cells from the 6th–9th passage were incubated in normal glucose (5 mM D-glucose, NG) or high glucose (20 mM D-glucose, HG) for 96 h [6]. A group of cells in 20 mM D-glucose were incubated in the presence of 100 μM L-homocysteine thiolactone hydrochloride (HG + Hcy group; Cat. No. S784036; Sigma-Aldrich, St. Louis, MO, USA) [26]. To investigate the effect of nitrosylation on mitochondrial fragmentation, cells incubated in high glucose, with or without homocysteine, also had a selective inhibitor of iNOS, 30 µM of N6-(1-iminoethyl)-L-lysine, dihydrochloride (NIL, Cat. No. 80310; Cayman Chemical, Ann Arbor, MI, USA; HG/NIL and HG + Hcy/NIL groups, respectively) [27].

**Figure 1:**
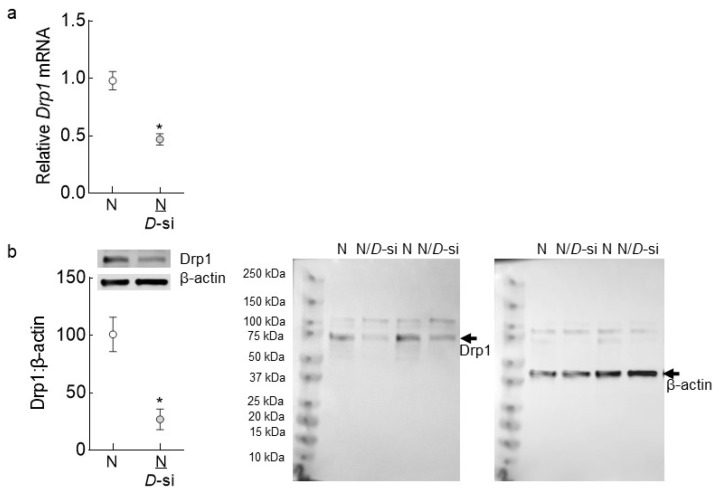
Transfection efficiency of *Drp1*-siRNA in retinal endothelial cells. The transfection efficiency *Drp1*-siRNA transfected HRECs was determined by quantifying Drp1 (**a**) gene transcripts (qRT-PCR, *β-actin* as a housekeeping gene) and (**b**) protein expression (Western blot, β-actin as a loading protein). The values are represented as mean ± SD from 3 or more samples, each per-formed in duplicate. N and N/*D*-si are untransfected or *Drp1*-siRNA transfected cells, respective-ly; * *p* < 0.05 vs. N.

A batch of HRECs from 6th to 7th passage was transfected with Silencer^TM^ Select *Drp1*-siRNA (Cat. No. 4390824, s19560, Thermo Fisher Scientific, Waltham, MA, USA) using Lipofectamine™ RNAiMAX transfection reagent (Cat. No. 13778150, Invitrogen™, Carlsbad, CA, USA) and then incubated in normal- or high-glucose medium for 96 h; nontargeting scrambled RNA was used as a control [4,6]. The transfection efficiency of *Drp1*-siRNA, as evaluated by quantifying its mRNA levels (by SYBR green-based quantitative real time PCR, qRT-PCR) and by its protein expression (by Western blot), was >50% (Figure 1). Each experiment was run with HRECs from the same batch and passage and was repeated 3–4 times. As an osmotic/metabolic control, cells incubated in 20 mM L-glucose (L-Gl), instead of 20 mM D-glucose, were also included in each experiment.

Mitochondrial fission: Mitochondrial fission was evaluated by live-cell microscopy using HRECs on coverslips that were incubated with 200 nM MitoTracker green FM (Cat. No. M7514, Thermo Fisher Scientific, Waltham, MA, USA) for 30 min [4,6]. The coverslips were rinsed with phosphate buffered saline (PBS) and then imaged using a ZEISS Apotome microscope (San Diego, CA, USA) at 40× objective. Each experimental condition had 6–8 cells/coverslip, and the experiment was repeated in three or more different cell preparations.

Nucleoids and mtDNA transcription: For quantifying nucleoids in the mtDNA, HRECs fixed with 100% methanol for 15 min were permeabilized with 0.5% triton X-100. After washing the cells with PBS, they were incubated in 1 mL 1X SYBR DNA gel stain (Ref S33102, Invitrogen) for 1 h at room temperature. The cells were then washed with PBS (2 × 5 min each) and mounted for imaging. Images were acquired using a 40× objective lens on a ZEISS microscope using a 488 nm wavelength filter and were analyzed with a ZEISS software module (Carl Zeiss, Inc., Chicago, IL, USA). Nucleoid foci were counted with an automated counting protocol by adjusting the threshold and keeping a size factor of 14 as selection criteria for the smallest foci in the ZEISS analysis module, as described previously [28].

RNA isolated from HRECs was used to quantify gene transcripts by SYBR green-based, real-time quantitative PCR (qRT-PCR) using gene-specific primers (Table 1), and β-actin was used as a housekeeping gene [4,6].

GTPase activity of Drp1: Activity of Drp1 was measured using the GTPase assay kit (DATG-200; Bioassay Systems, Hayward, CA, USA). Protein (200μg) was lysed in the lysis buffer (50 nM HEPES, pH7.5, 120 mM NaCl, 5 mM EDTA, 10 mM sodium pyrophosphate, 50 mM NaF, 1 mM Na3VO4, 1% Triton X-100 and protease inhibitors) and immunoprecipitated using 3μg Drp1 antibody (Cat. No. sc-271583, Santa Cruz Biotechnology, Santa Cruz, CA, USA). The immunoprecipitate was incubated with Protein A/G Plus agarose beads (suspended in the lysis buffer), and the beads were then washed with the wash buffer (50 mM Tris, pH 7.5, 2.5 mM MgCl_2_, 0.02% β-mercaptoethanol). After incubating the beads with 0.5 mM GTP at room temperature for 30 min, phosphate released by the conversion of GTP into GDP was quantified at 620 nm [29].

Mitochondrial translocation of Drp1: Immunofluorescence microscopy was performed using Drp1 antibodies (Cat. No. ab184247, Abcam, Cambridge, MA, USA; 1:200 dilution) and Tom20 as a mitochondrial marker (Cat. No. MABT166, EMD Millipore, Temecula, CA; 1:200 dilution). Secondary antibodies for Drp1 and mitochondria included Alexa Fluor-488 (green) conjugated anti-rabbit (Cat. No. A11008, Molecular Probes–Life Technologies, Grand Island, NE, USA; 1:500 dilution) and Texas red-conjugated anti-mouse (Cat. No. TI200, Vector Laboratories, Burlingame, CA, USA; 1:500 dilution), respectively. After mounting the immunolabelled cells in DAPI-containing (blue) Vectashield mounting medium (Vector Laboratories), they were imaged using a 63× oil objective lens on a ZEISS microscope. Pearson’s correlation coefficient between Drp1 and Tom20 was calculated using the colocalization Zeiss software module [28].

Capillary cell apoptosis: Apoptosis was quantified by employing a Cell Death Detection ELISA PLUS kit from Roche Diagnostics (Cat. No. 11774425001, Indianapolis, IN, USA) as described previously [18]. The results were calculated as percentage, considering the values obtained from cells in normal glucose as 100%.

Post-translation modification of Drp1: Nitrosylation of Drp1 was measured using 2,3-diaminonaphthalene, as described by others [30]. In brief, the lysate was immunoprecipitated by 3 μg Drp1 antibody, and, after rinsing the immunoprecipitate with PBS, the sample was incubated with 200µM HgCl_2_ and 200 µM 2,3-diaminonaphthalene for 30 min in the dark. Nitic oxide released from S-nitrosylated Drp1 was followed at 364 nm (excitation) and 430 nm (emission) wavelengths, reflecting the conversion of 2,3-diaminonaphthalene to fluorescent 2,3-naphthyltriazole.

Statistical analysis: Graph Pad Prism (San Diego, CA, USA), was used for statistical analysis, and the data are presented as mean ± SD. One-way ANOVA followed by Dunn’s *t*-test were employed for group comparison. A *p* < 0.05 was considered significant.

## 3. Results

**Figure 2:**
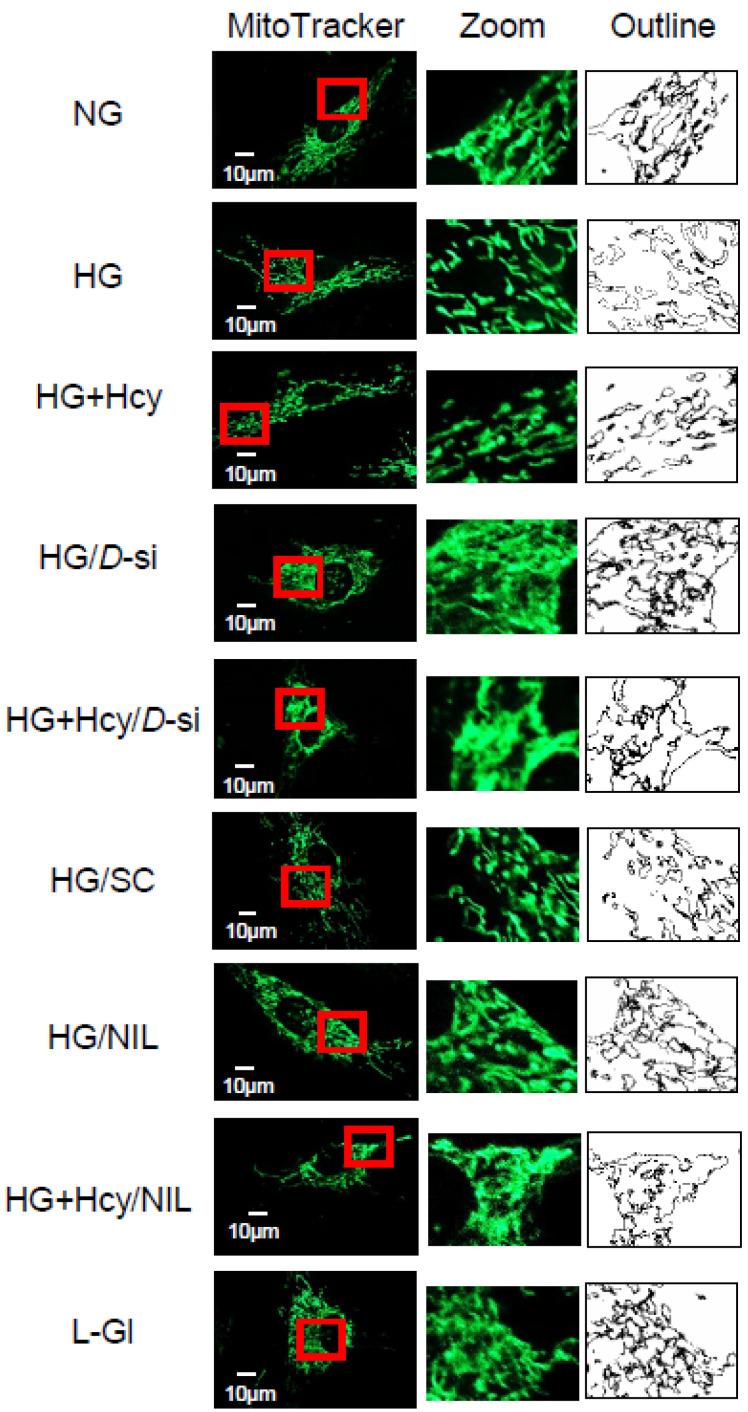
Effect of homocysteine on glucose-induced mitochondrial fission. Live-cell microscopy was performed on HRECs plated on coverslips using 200 nM MitoTracker green dye. The coverslips were imaged under a ZEISS Apotome microscope using a 40× objective. Enlarged area inside the box is shown in the middle panel, and the bare outlines, drawn to illustrate the morphology, are in the right panel. The image is representative of 3–4 different experiments, with each experiment performed in 6–8 cells: NG and HG, cells in normal and high glucose, respectively; HG + Hcy, cells in high glucose and homocysteine; HG/*D*-si and HG + Hcy/*D*-si, *Drp1*-siRNA transfected cells in high glucose or high glucose + homocysteine, respectively; HG/SC, nontargeting scrambled RNA transfected cells in high glucose; HG/NIL and HG + Hcy/NIL, cells in the presence of NIL in high glucose or high glucose + homocysteine, respectively; L-Gl, 20 mM L-glucose.

Effect of homocysteine on mitochondrial fission: Homocysteine affects normal mitochondrial structure and function, and in diabetic retinopathy, mitochondrial dynamics is impaired [6,20]. As reported previously [6], compared to cells in normal glucose, high glucose increased mitochondrial fission, which was further exaggerated by supplementation of homocysteine (HG + Hcy group; *p* < 0.05 vs. HG). Cells incubated in 20 mM L-glucose instead of 20 mM D-glucose had a similar degree of mitochondrial fragmentation as the cells in the NG group (Figure 2).

**Figure 3:**
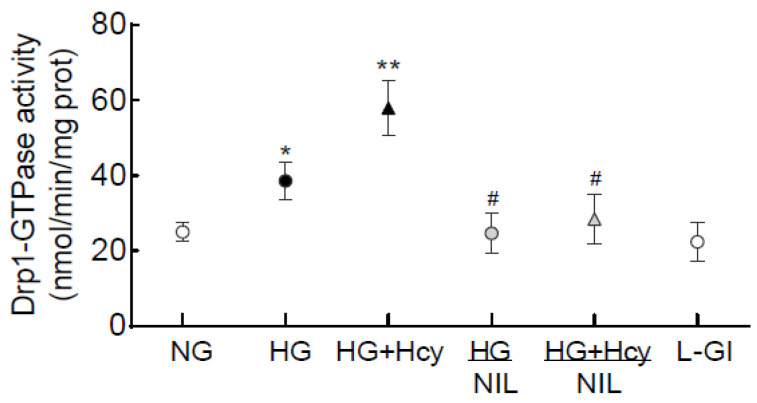
Effect of homocysteine on GTPase activity of Drp1. GTPase activity of Drp1 was measured in the Drp1-immunoprecipitated samples, and the release of phosphate was quantified spectrophotometrically. The values are represented as mean ± SD obtained from 4–5 different cell preparations with each measurement made in duplicate. * *p* < 0.05 vs. NG; ** *p* < 0.05 vs. HG; # *p* > 0.05 vs. NG.

Effect of homocysteine on Drp1 activity: GTPase activity of Drp1 is essential for the fission process [8,11]; consistent with mitochondrial fission, the activity of Drp1 was further increased in HRECs incubated in glucose medium supplemented with homocysteine (*p* < 0.05 vs. HG group; Figure 3). However, 20 mM L-glucose, instead of 20 mM D-glucose, had no effect on Drp1 activity, and the values in NG and L-Gl groups were not different from each other (*p* > 0.05).

**Figure 4:**
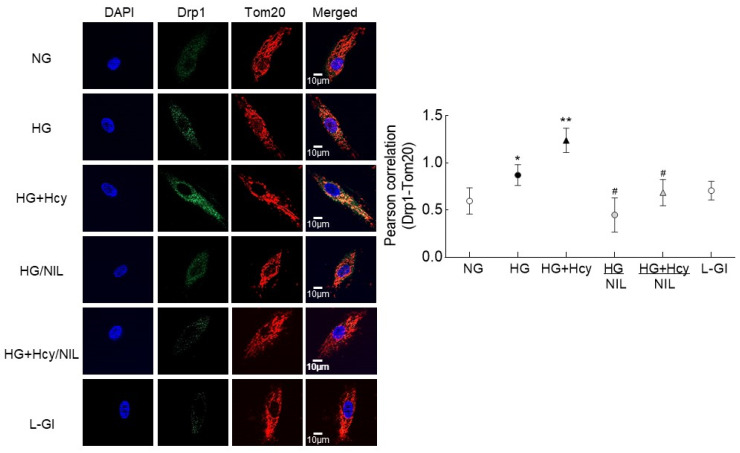
Mitochondrial translocation of Drp1. Mitochondrial localization was determined by immunofluorescence technique using Alexa Fluor–488 (green) conjugated secondary antibody for Drp1 and Texas red conjugated for the mitochondrial marker Tom20. DAPI-containing (blue) Vectashield mounting medium was used for mounting the cells, and the cells were imaged using a 63× oil objective lens on a ZEISS microscope. Pearson’s correlation coefficient between Drp1 and Tom20 was calculated using the colocalization ZEISS software module. The values in the graphs are presented as mean ± SD obtained from 3–4 different cell preparations. NG, cells in normal glucose; HG and HG + Hcy, cells in high glucose and high glucose + homocysteine respectively; HG/NIL and HG + Hcy/NIL, cells in high glucose containing NIL in the presence or absence of homocysteine, respectively; L-Gl, 20 mM L-glucose; * *p* < 0.05 compared to NG; ** *p* < 0.05 compared to HG; # *p* > 0.05 compared to NG.

Homocysteine and translocation of Drp1 to the mitochondria: Movement of the cytosolic Drp1 into the mitochondria is important to promote mitochondrial fission [31]; as shown in Figure 4, homocysteine supplementation further increased glucose-induced mitochondrial accumulation of Drp1, as indicated by increased Drp1-Tom20 colocalization. This was further confirmed by an increased Pearson correlation coefficient between Drp1 and Tom20 in the cells in HG + Hcy group compared to HG group (Figure 4).

**Figure 5:**
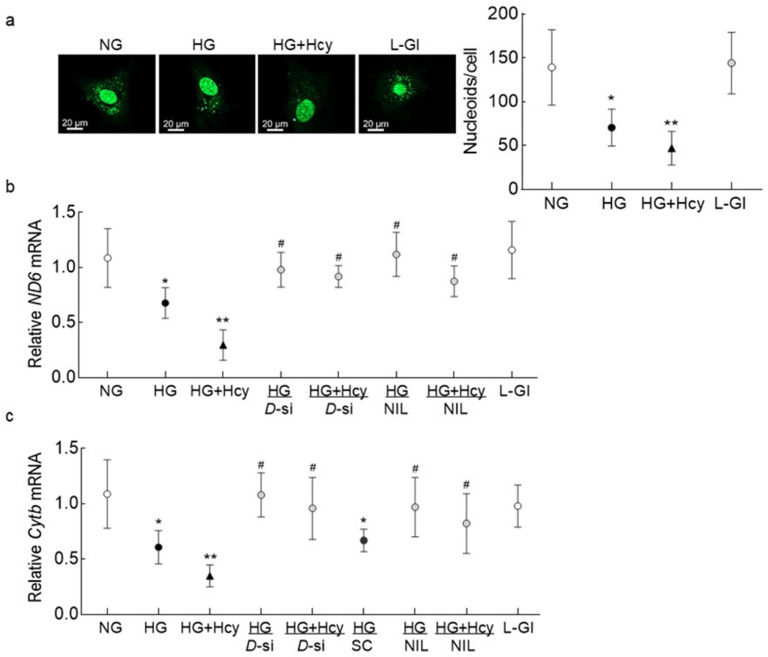
Effect of homocysteine on mitochondrial nucleoids and transcription of mtDNA. (**a**) Nucleoids were quantified using SYBR DNA gel stain, and the images were acquired using a 40× objective. The accompanying graph has values represented as mean ± SD obtained from 30–40 cells in each group. Gene transcripts of mtDNA-encoded (**b**) *ND6* and (**c**) *Cytb* were quantified by qRT-PCR using *β-actin* as a housekeeping gene. The values are represented as mean ± SD obtained from 3–4 different cell preparations with each measurement made in triplicate: NG and HG, cells in normal and high glucose, respectively; HG + Hcy, cells in high glucose and homocysteine; HG/*D*-si and HG + Hcy/*D*-si, *Drp1*-siRNA transfected cells in high glucose or high glucose + homocysteine, respectively; HG/SC, nontargeting scrambled RNA transfected cells in high glucose; HG/NIL and HG + Hcy/NIL, cells in the presence of NIL in high glucose or high glucose + homocysteine, respectively; L-Gl, 20 mM L-glucose; * and ** *p* < 0.05 vs. NG and HG, respectively; # *p* > 0.05 vs. NG.

Homocysteine and mitochondrial damage: To further determine the role of Drp1 in homocysteine-mediated mitochondrial damage, nucleoids in the mtDNA were evaluated. High glucose significantly reduced the number of nucleoids, which was further decreased when homocysteine was introduced in the high-glucose medium (Figure 5a). The values obtained from cells in HG and HG + Hcy groups were significantly different from each other and were also different from the cells in NG or L-Gl groups (*p* < 0.05). Since damage to the mtDNA impairs its transcription, gene transcripts of mtDNA-encoded *ND6* of complex I and *CytB* of complex III were quantified; gene transcripts of both *ND6* and *CytB* were significantly decreased in cells incubated in HG + Hcy compared to cells in HG (Figure 5b,c).

**Figure 6:**
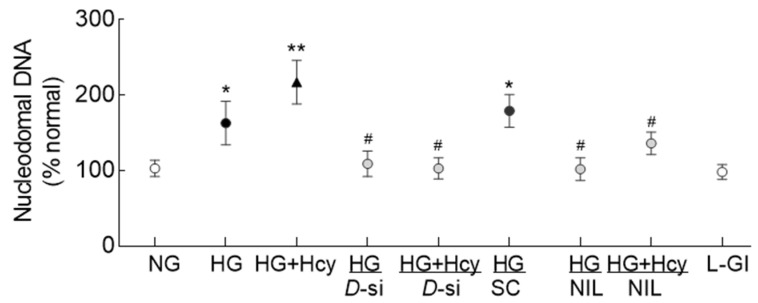
Homocysteine and endothelial cell apoptosis. Apoptosis of HRECs was quantified using a Cell Death Detection ELISA PLUS kit from Roche Diagnostics, and the values obtained from cells in normal glucose were considered as 100: NG and HG, cells in normal and high glucose, respectively; HG + Hcy, cells in high glucose and homocysteine; HG/*D*-si and HG + Hcy/*D*-si, *Drp1*-siRNA transfected cells in high glucose or high glucose + homocysteine, respectively; HG/SC, nontargeting scrambled RNA transfected cells in high glucose; HG/NIL and HG + Hcy/NIL, cells in the presence of NIL in high glucose or high glucose + homocysteine, respectively; L-Gl, 20 mM L-glucose; * and ** *p* < 0.05 vs. NG and HG, respectively; # *p* > 0.05 vs. NG.

Mitochondrial damage is intimately associated with cell apoptosis [32]; Figure 6 shows that although glucose exposure increased cell apoptosis by ~75%, addition of homocysteine further exacerbated glucose-induced cell apoptosis. Values obtained from cells in HG + Hcy were significantly higher compared to those in the HG group (*p* < 0.05), but incubation of cells in 20 mM L-glucose had no effect on apoptosis, and the values were similar to those obtained from cells in the NG group.

Role of Drp1 in exacerbation of mitochondrial damage in high glucose -high homocysteine medium: To confirm the role of Drp1 in homocysteine-mediated increased mitochondrial fission in a hyperglycemic milieu, cells transfected with *Drp1*-siRNA were employed. As shown in Figure 2, *Drp1*-siRNA transfected cells incubated in high glucose + homocysteine medium (HG + Hcy/*D*-si group) had less mitochondrial fragmentation compared to untransfected cells in the same medium. Similarly, transfection with *Drp1*-siRNA also prevented decrease in the gene transcripts of mtDNA-encoded *ND6* and *CytB* and decreased the cell apoptosis experienced by the cells in high glucose, both in the presence or absence of homocysteine. However, transfection with nontargeting scrambled RNA had no effect on glucose-induced decrease in *Cytb* mRNA and increase in cell apoptosis (Figure 5b and Figure 6).

**Figure 7:**
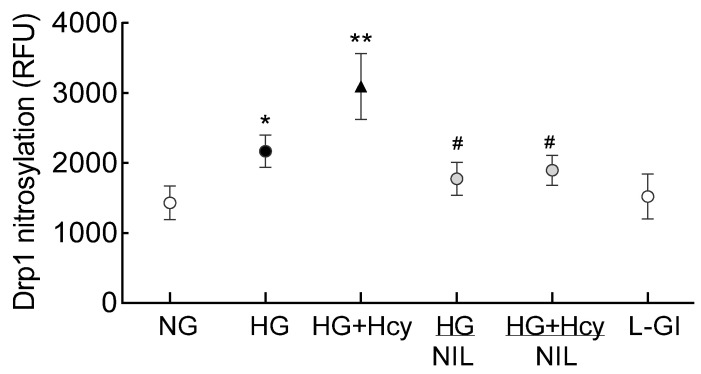
Effect of homocysteine on Drp1 nitrosylation. Drp1 nitrosylation was determined by following the conversion of 2,3-diaminonaphthalene to fluorescent 2,3-naphthyltriazole. The values presented are mean ± SD from 3–4 different cell preparations with each measurement made in duplicate: NG and HG, cells in normal and high glucose respectively; HG + Hcy, cells in high glucose + homocysteine; HG/NIL and HG + Hcy/NIL, cells in the presence of NIL in high glucose or high glucose + homocysteine, respectively; L-Gl, 20 mM L-glucose; * and ** *p* < 0.05 vs. NG and HG, respectively; # *p* > 0.05 vs. NG.

Nitrosylation of Drp1: Post-translational modifications of Drp1 play a major role in its activation [30]; the effect of high glucose on nitrosylation of Drp1 was determined. As shown in Figure 7, nitrosylation of Drp1 was significantly increased by high glucose, and this was further exacerbated when the high-glucose medium was supplemented with homocysteine. Inhibition of nitrosylation by NIL prevented glucose- and glucose + homocysteine-induced increase in nitrosylation of Drp1, and the values obtained from cells in HG/NIL and HG + Hcy/NIL groups were not different from each other (*p* > 0.05). In the same cell preparation, Drp1 activation and its translocation inside the mitochondria, induced by high glucose or by glucose + homocysteine, were also prevented by inhibition of nitrosylation (Figure 3 and Figure 4). The gene transcripts of *ND6* and *CytB* and cell apoptosis in the cells incubated in high glucose/NIL, both in the presence or absence of homocysteine, were also comparable to those obtained from cells in normal glucose (Figure 5 and Figure 6).

## 4. Discussion

Diabetes damages retinal mitochondria, impairing their structural, functional and genomic stability and disturbing the balance between their fusion and fission [2,3,4]. Diabetic patients also have elevated circulating levels of homocysteine, and plasma homocysteine is considered as a risk factor for increased risk of diabetic retinopathy [7,15,18]. In addition, higher homocysteine levels are observed in the retina from experimental models of diabetic retinopathy and from human donors with established diabetic retinopathy [7,18]. Here, our exciting data show that homocysteine exacerbates glucose-induced Drp1 translocation to the mitochondria and mitochondrial fragmentation in retinal endothelial cells, further decreasing the number of protective nucleoids in the mtDNA and the transcription of mtDNA-encoded genes critical for the functioning of the electron transport chain system. Cell apoptosis is also exacerbated, and regulation of *Drp1* by its siRNA prevents glucose + homocysteine-induced mitochondrial fragmentation and cell apoptosis. Furthermore, high glucose increases nitrosylation of Drp1, which is also aggravated by homocysteine, and prevention of nitrosylation by an inhibitor of NO protects Drp1 activation and mitochondrial fragmentation and capillary cell apoptosis. Thus, by facilitating Drp1 movement in the mitochondria and increasing its GTPase activity, elevated homocysteine in a hyperglycemic environment further shifts mitochondrial dynamics towards increased fragmentation, which increases capillary cell apoptosis, ultimately accelerating the development of diabetic retinopathy.

A highly regulated mitochondrial dynamic is imbalanced in diabetes; while fission machinery is upregulated, fusion machinery is downregulated, and this results in fragmented mitochondria [6]. Here, we show that the combination of high glucose and homocysteine further exacerbates mitochondrial fragmentation; homocysteine further increases glucose-induced translocation of Drp1 to the mitochondria and increases its GTPase activity. Furthermore, *Drp1*-siRNA ameliorates Drp1-activated mitochondrial translocation and fragmentation, confirming the role of Drp1 in homocysteine-mediated exacerbation of mitochondrial fragmentation in a hyperglycemic milieu. In support, homocysteine has been shown to damage the mitochondrial fusion–fission process [19], and mitochondria from mice lacking cystathionine-β-synthase are smaller in size and have increased mitochondrial fission compared to mitochondria from wildtype mice [20].

Mitochondrial DNA is small, with ~16K base pairs which encode for only 13 proteins, and all of these proteins are critical in the functioning of the electron transport chain system. It is in close proximity to the oxygen-producing respiratory chain, and, unlike nuclear DNA, mtDNA lacks protective histones. However, mtDNA is packaged with proteins, the nucleoids, and each nucleoid can have hundreds to thousands copies of mtDNA [33]. These nucleoids provide a stable environment and protect mtDNA from the damaging free radicals; these nucleoids are not randomly distributed in the mitochondrial matrix but are often found close to the mitochondrial cristae or wrapped around a cristae-like structure [34]. Furthermore, nucleoids have ordered, toroidal structures, which depend on the physiological state of cells; e.g., nucleoids reorganize in stationary phase cells and their morphology undergoes massive transformation [35,36]. Here, we show that glucose-induced decrease in nucleoids is further worsened by homocysteine in the medium. Mitochondrial dynamics and mtDNA are intimately associated, as the mitochondrial fusion–fission process plays an important role in the elimination of the irreparably damaged mtDNA. Normal mitochondrial dynamics, in addition to being critical in the redistribution of mitochondria to sites requiring high energy production, are also important in the formation of new mitochondria and repair of defective mitochondrial DNA through mixing [5,8,11,37]. Moreover, the distribution of mtDNA throughout the mitochondria is tightly linked with their continuous fusion and fission, and fusion promotes complementation of mtDNA between two mitochondria [38]. Our results show that homocysteine, in addition to reducing nucleoids, further decreases the transcripts of mtDNA-encoded *Cytb* and *ND6*, suggesting the role of homocysteine in impaired mtDNA transcription is closely associated with a compromised electron transport chain system and dysfunctional mitochondria [1,2]. Furthermore, regulation of *Drp1* by its siRNA ameliorates both glucose- and glucose + homocysteine-induced impaired transcription of mtDNA, as shown by similar gene transcripts of *Cytb* or *ND6* in *Drp1*-siRNA transfected cells exposed to high glucose or high glucose + homocysteine and cells in normal glucose, suggesting a role of Drp1 in maintaining mtDNA stability. In support, changes in mitochondrial dynamics are also implicated in mtDNA damage, and a close proximity between mtDNA and the sites of Drp1-dependent mitochondrial fission is observed in many mammalian cells [39,40].

Posttranslational modifications play an important role in altering activities of many enzymes [22,41], and a diabetic environment is shown to regulate many metabolic and molecular pathways by facilitating different posttranscriptional modifications [42,43]. Nitric oxide, in addition to reacting with superoxide radicals to form peroxynitrite, can also react with sulfhydryl (thiol) groups of proteins and form *S*-nitrosothiols. Nitrosylation is a ubiquitous post-translational modification and is responsible for a broad spectrum of biological functions; in endothelial cells, it is shown to affect cellular metabolic processes and regulate vascular functions including inflammation and apoptosis [44,45]. This redox-based post-translational modification can regulate cysteine residues at the active site or induce allosteric changes of protein structures, and it is implicated in both physiological and pathological conditions; e.g., aberrant nitrosylation increases the GTPase activity of Drp1, resulting in excessive mitochondrial fragmentation [21,22,23]. Here, our results show that high glucose increases Drp1 nitrosylation, which is exacerbated by homocysteine; in support, excessive accumulation of nitrosative stress triggers abnormal mitochondrial morphology in brains of neurodegenerative patients [30]. We demonstrate that inhibition of increased NO production, in addition to inhibiting nitrosylation of Drp1 and its activity, also ameliorates mitochondrial fragmentation and mtDNA damage-induced apoptosis resulting from hyperglycemia and hyperhomocysteinemia. This suggests that nitrosative stress could have a major role in mitochondrial fragmentation in diabetic retinopathy; this is supported by our previous study showing increased accumulation of peroxynitrite in the retinal microvasculature, the site of histopathology of diabetic retinopathy, in diabetes [46]. Furthermore, nitrosylation of caveolin is implicated in the breakdown of the outer blood–retinal barrier in diabetes [47], and that of Drp1 is observed in several neurodegenerative diseases, including Parkinson’s disease and Huntington’s disease [48,49].

We recognize that our study is focused on the role of nitrosylation, and the role of other modifications, including phosphorylation and ubiquitination, in the regulation of Drp1 activity in diabetic retinopathy, however, cannot be ruled out. Moreover, we have focused on Drp1, but a balance between mitochondrial fusion–fission is critical for homeostasis, and the role of homocysteine in altering mitochondrial dynamics via affecting Mfn2 remains to be investigated.

## 5. Conclusions

In summary, we demonstrate the role of homocysteine in Drp1-mediated mitochondrial damage in the development of diabetic retinopathy. Although Drp1 activity and its mitochondrial translocation are increased in hyperglycemic milieu, addition of homocysteine further activates Drp1 and increases mitochondrial fragmentation. The possible mechanism for homocysteine-mediated mitochondrial fragmentation and damage in diabetes appears to be increased nitrosylation of Drp1. Thus, our results clearly show the importance of regulating homocysteine levels, in addition to sugar levels, for a diabetic patient to protect their retinal mitochondrial from being damaged and to prevent/slow down diabetic retinopathy.

## Figures and Tables

**Table 1:** Primer sequences for human genes.

Primer	Sequence
*Drp1*	Fwd-GAA GGA GGC GAA CTG TGG GC
	Rev-GCA GCT GGA TGA TGT CGG CG
*ND6*	Fwd-CCAAGACCTCAACCCCTGAC
	Rev-GGTGTGGTCGGGTGTGTTAT
*Cytb*	Fwd-TCA CCA GAC GCC TCA ACC GC
	Rev-GCC TCG CCC GAT GTG TAG GA
*β-Actin*	Fwd-AGC CTC GCC TTT GCC GAT CCG
	Rev-TCT CTT GCT CTG GGC CTC GTCG

## Data Availability

Data is contained within the article.
